# Portable, non-destructive colorimetry and visible reflectance spectroscopy paired with machine learning can classify experimentally heat-treated silcrete from three South African sources

**DOI:** 10.1371/journal.pone.0266389

**Published:** 2022-04-08

**Authors:** John K. Murray, Simen Oestmo, Andrew M. Zipkin

**Affiliations:** 1 School of Human Evolution and Social Change, Arizona State University, Tempe, AZ, United States of America; 2 Institute of Human Origins, Arizona State University, Tempe, AZ, United States of America; 3 African Centre for Coastal Palaeoscience, Nelson Mandela University, Gqeberha, South Africa; New York State Museum, UNITED STATES

## Abstract

The objective of this study was to determine if visible reflectance spectroscopy and quantitative colorimetry represent viable approaches to classifying the heat treatment state of silcrete. Silcrete is a soil duricrust that has been used as toolstone since at least the Middle Stone Age. The ancient practice of heat treating silcrete prior to knapping is of considerable interest to paleolithic archaeologists because of its implications for early modern human complex cognition generally and the ability to manipulate the material properties of stone specifically. Here, we demonstrate that our quantitative, non-invasive, and portable approach to measuring color, used in conjunction with *k*-Nearest Neighbors “lazy” machine learning, is a highly promising method for heat treatment detection. Traditional, expert human analyst approaches typically rely upon subjective assessments of color and luster and comparison to experimental reference collections. This strongly visual method can prove quite accurate, but difficult to reproduce between different analysts. In this work, we measured percent reflectance for the visible spectrum (1018 variables) and standardized color values (CIEL*a*b*) in unheated and experimentally heat-treated silcrete specimens from three sources in South Africa. *k-*NN classification proved highly effective with both the spectroscopy and colorimetry data sets. An important innovation was using the heat treatment state predicted by the *k*-NN model for the majority of replicate observations of a single specimen to predict the heat treatment state for the specimen overall. When this majority voting approach was applied to the 746 individual observations in this study, associated with 94 discrete silcrete flakes, both spectroscopy and colorimetry *k*-NN models yielded 0% test set misclassification rates at the specimen level.

## Introduction

Since the realization that heat treatment alters the flaking parameters of silicate rocks such as chert, jasper, and silcrete, researchers have conducted heating experiments to better understand the relationship between the physical and chemical changes of the stone and its flaking properties [1–11]. Generally, heat-treated siliceous stone becomes more lustrous and smoother [12–16] and sometimes darker and redder [13, 17–24]. Traditionally, analyst observation of these visual and tangible changes was the most common way to identify heat treatment in archaeological assemblages. Schmidt [13] has shown that with the aid of a reference collection, the visual identification of heat-treated silcrete can yield up to ~92% accuracy. Although necessary for heat treatment analyses, visual methods are typically dependent upon expert knowledge to perform at such a high level which hinders meta-analyses because there is not a standard operating procedure for observing silcrete that specifies parameters like lighting conditions. Considering the behavioral implications of heat treatment technology [12, 25, 26], our study introduces a quantitative method that can be used to bolster our ability to identify heat-treated silcrete and increase our confidence in assignments of heat treatment using visual methods.

This study seeks to determine if the heat treatment condition of silcrete can be predicted as a binary state (yes/no) using machine learning models built with quantitative colorimetry and reflectance spectroscopy data. We investigate multiple approaches to data processing and predictive classification and assess the effectiveness of the resulting models in order to provide guidance for other researchers seeking to customize and deploy this system for their own study areas. Further, we explore how color varies within and between three archaeologically relevant silcrete sources near Pinnacle Point, South Africa. We believe that quantitatively characterizing silcrete color using a standardized instrumental approach can improve the accuracy, reproducibility, and reliability of studies seeking to identify heat treatment in the archaeological record. This builds on other approaches that aim to reduce subjectivity in heat treatment analysis [13–15, 27, 28]. We analyzed experimentally heat-treated and unheated silcrete samples to create a reference dataset of observations for five colorimetry variables from the CIE (Commission International d’Eclairage) L*a*b* color space for each source [29]. Additionally, we explore the effectiveness of higher resolution reflectance spectroscopy measurements of over 1000 wavelength variables across the visible spectrum for identifying heat-treated silcrete with. To test whether the colorimetric and spectroscopic data collected could be used to differentiate heated and unheated silcrete, we built *k-*Nearest Neighbor predictive classification models for both L*a*b* and percent reflectance variables.

Our experiment was limited to silcrete from close to the town of Mossel Bay located on the southern coast of the Western Cape province of South Africa; however, our methods have the potential to be used on silcrete from other regions and even for multiple raw material types because similar color changes are documented in flint using colorimetry [22]. The data collection protocol described here is field-portable and non-destructive, and the statistical analyses were carried out using commercially available software. Our approach can be used as a stand-alone method to identify heat-treated silcrete or be used in conjunction with other methods to verify treatment predictions with an additional line of evidence [13–16, 28, 30].

## Background

### Heat treatment technology

When silcrete is heated, it undergoes physical and chemical changes that lead to a higher quality raw material due to an increase in hardness and reduction in fracture toughness which makes it easier to flake [5, 6, 9–12, 31–34]. In addition to the change in quality of the toolstone for knapping, heat treatment has other functional benefits such as an increase in edge durability and edge sharpness [35, 36], an increase in the cutting edge to mass ratio [37], and heat-treated material is more amenable to pressure flaking for retouch or blade/micro-blade production [38–41]. Heat treatment also causes silcrete to change in color, increase in glossiness, and decrease in surface roughness [12–14]. Ultimately, heat treatment technology allowed Middle Stone Age (MSA) humans to improve the quality of silcrete toolstone to make better, and possibly smaller, tools [36, 37] while increasing the net-return rate of procuring silcrete raw material [35].

There are other factors that must be considered which may influence heat treatment. First, research suggests that the size of the nodule being heated influences the likelihood that it will successfully heat-treat without fracturing [42]. Additionally, silcrete is highly variable–both intra- and inter-source–and each source responds differently to heat treatment [43, 44]. This suggests that substantial comprehension of raw material quality and the mechanics of controlling fire would have been required to successfully heat-treat silcrete.

### Identifying heat-treated silcrete

There are multiple approaches to identifying heat-treated siliceous stone which include both qualitative and quantitative methods. Almost 40 years ago Olausson and Larrson [45] highlighted 11 different means of testing whether flint had been heat-treated and the number of available approaches has only increased since, as illustrated by the references cited below. The most common ways to identify heat-treated silcrete are through observing physical changes to the stone, such as color change, a reduction in surface roughness, an increase in luster, and the presence of crazing, potlids, tempering residue, and heat-induced non-conchoidal (HINC) fractures [46]. Visual indicators of heat treatment have been shown to be accurate for multiple silcrete types from the West Coast of South Africa [13] and Australia [27], but due to the low specimen count for each type of silcrete in their reference collection, it is unclear how inter-/intra-source and intra-specimen variability effects its accuracy. These qualitative measures of heat treatment are helpful because they allow the researcher to sample a large number of artifacts and select particular specimens for further analysis with quantitative instrumental methods such as Raman or near-infrared spectroscopy [11, 16, 28, 47–49] or thermoluminescence [12, 50, 51]. Due to considerable variation in how silcrete from different sources responds to heat treatment [43, 44], the visual approach requires a large reference collection and expert knowledge to be viable.

Recently, lithic heat treatment has seen a significant amount of attention due to the potential connections with early human cognition and social learning. This research has primarily focused on developing methods to identify heat treatment that are both non-destructive and quantitative [10, 14–16, 27, 28, 30]. Two of these approaches exploit changes in surface roughness to detect heat treatment using replica-tape [13, 27] and 3D microscopy [14–16]. Agam and colleagues [28] apply UV Raman spectroscopy to determine the temperature at which archaeological specimens from Qesem Cave, Israel were heated. Santaniello *et al*. [30] show that it is possible to identify heat-treated chert based on density measurements using an Archimedes balance and an experimental reference curve.

The use of instrumental methods to collect quantitative data has facilitated the application of increasingly complex statistical and machine learning approaches to data analysis. For example, two previously described studies apply machine learning algorithms [28] and Bayesian statistics [14] to increase the reliability of their results. Murray and colleagues [14] developed a Bayesian model that can assign each sample a level of treatment with an associated probability of assignment (*i*.*e*., heated or unheated) which improves the accuracy with which we can identify heat treatment in the record. Agam and colleagues [28] utilize machine learning (logistic regression, linear support vector machines, and neural networks) to develop a temperature-estimation model and better understand the conditions under which certain lithic materials were heated. They argue that blades were consistently heated at lower temperatures compared to flakes, which suggests that by 300,000 years ago Levantine hominins had a high degree of technological control over fire.

### Color change and heat treatment

Color change in heat-treated stone has been documented using multiple approaches that include both qualitative and quantitative methods. One of the simplest ways that researchers have described color change in heat-treated stone is using broad, categorical color assignments such as “black” or “red” [19, 24, 52]. Another approach that is more standardized, but still categorical, is the use of Munsell color system to describe the color of heated and unheated stone [18, 21, 24]. Additionally, researchers have documented color change using RGB values taken from photographs of stone tools [20]. The RGB approach is useful because it is quantitative and can be standardized within the sRGB color space. Schmidt and colleagues [20] used this approach to describe color change in heat-treated silcrete from Botswana and South Africa across various temperatures. Lastly, colorimetry from direct measurement of toolstone–specifically in the CIE L*a*b* color space–has been applied to better understand thermal induced changes in flint [22], fire modified rock [53], and archaeological sediments [54]. The CIE L*a*b* color space consists of three variables: L*, a*, and b*. L* is the lightness value of the color where 0 is black and 100 is white (*i*.*e*., the lower the value the darker the color). a* is the relative green-red component of the color where negative values are more green and positive values are more red. Likewise, b* is the measure of a color’s blue-yellow component where negative values are more blue and positive values are more yellow. Two additional measurements, hue-angle and chroma, may be derived from L*a*b* color space. Hue-angle is a numerical value that corresponds to an angular position in the CIE L*a*b* color wheel. Chroma is a measurement of the quality of the color’s saturation, purity, and intensity. Hue-angle, when combined with chroma, recapitulates the same information captured by the L*, a*, and b* variables in an RGB-based two variable alternative to the tristimulus (i.e., standardize color system with three variables) CIE L*a*b* color system.

Fiers and colleagues [22] calculate the Δ values for L*, a*, and b* to describe color changes in flint across various temperatures. Further, they utilize ΔE, which calculates overall color change between unheated and heated flint with the CIE L*a*b* 1976 color difference formula. The first phase of our study adopts the approach of Fiers and colleagues [22] to better understand variation in color change in silcrete from three sources in South Africa. We then use these data to statistically discriminate unheated silcrete from heat-treated silcrete. Specifically, we utilize the L*a*b* colorimetry values to build our statistical models, rather than the Δ values because, as Fiers and colleagues [22] point out, it is not possible to collect data on heat-treated archaeological artifacts in their unheated form to calculate the color change values. In the second phase of our study, we expand on this method by utilizing the nanometer-scale visible light reflectance spectroscopy data that underlie the three colorimetry variables and were collected simultaneously with them. The spectral reflectance data provide us with a more nuanced measure of color and vastly increased number of variables for statistical exploration.

We think that colorimetry and reflectance spectroscopy have the potential to be important tools for identifying heat treatment in the archaeological record. Modular UV-Vis-NIR spectrometers such as the model that we use here are portable and relatively inexpensive compared to instruments used in other types of vibrational spectroscopy. Some models can even interface with smartphones and tablets for data collection, facilitating the study of heat treatment in the contexts of landscape surveys or cultural resource management archaeology. Dedicated colorimeters that are capable of measuring CIE L*a*b* and other color space variables but not reflectance spectroscopy variables may eventually prove to be an even more field- and user-friendly option with sufficient method development and validation.

## Materials and methods

### Heat treatment experiments

The heat treatment process used for this research is described in detail by Murray and colleagues [14] and Oestmo [35] so only summary information is provided here. Silcrete raw material samples were collected by Dr. Kyle Brown during surveys in the Mossel Bay region on the Western Cape of South Africa from three primary outcrops, D9-1, E3-1, and I14-2 (referred to as D9, E3, and I14 throughout the paper), within ~80 km of the Pinnacle Point archaeological site complex [55, 56]. These sources are very fine-grained matrix supported silcretes [14] which are common in the archaeological record [5, 6, 57–59]. Many of these samples contain scattered quartz grains throughout the matrix. Source I14 is a part of the Bokkeveld formation of the Cape Town supergroup whereas sources D9 and E3 are of the Grahamstown formation. The nodules collected from all three sources are variable in shape and range from rectangular to globular in shape and angular to sub-angular in roundness [14]. The silcrete employed for this study derives from a dedicated research assemblage of toolstone maintained at Arizona State University for method development. The silcrete specifically used in our experiment was originally collected, heat-treated, and knapped by Kyle Brown and Simen Oestmo [35].

Silcrete nodules from each source were cut to create paired 7 x 7 x 15 cm blocks (*i*.*e*., to yield one heated and one unheated block from the same nodule). A total of 16 blocks were precut into similar sized and shaped pieces to minimize variance for the following heat-treatment procedure and flaking experiment [35]. Source D9 yielded three heated and three unheated blocks, I14 a single pair of one heated and one unheated, and E3 produced four heated and four unheated blocks. The eight silcrete blocks designated for heat treatment were heated using temperature and duration specifications outlined by Brown *et al*. [12]. Samples were heated in an electric kiln fitted with an external J-Kem programmable temperature controller (Model 360/Timer-K) and Digi-Sense DualLogR thermocouple thermometer. The temperature of the furnace was ramped to 350°C over five hours. This temperature was held constant for 12 hours and then dropped slowly to 40°C. The cooled blocks were subsequently knapped to produce the flakes used here for color analysis [35]. We think that 350°C is a good temperature for this reductionist model because previously published papers using different methods to measure color have shown that around 300°-350°C silcrete reaches maximum color change and begins to decline in these changes above these temperatures [20]. Further, artifacts analyzed from the south coast of South Africa, specifically from Pinnacle Point and Blombos, have been heated to temperatures between 300°-400°C [12].

**Fig 1:**
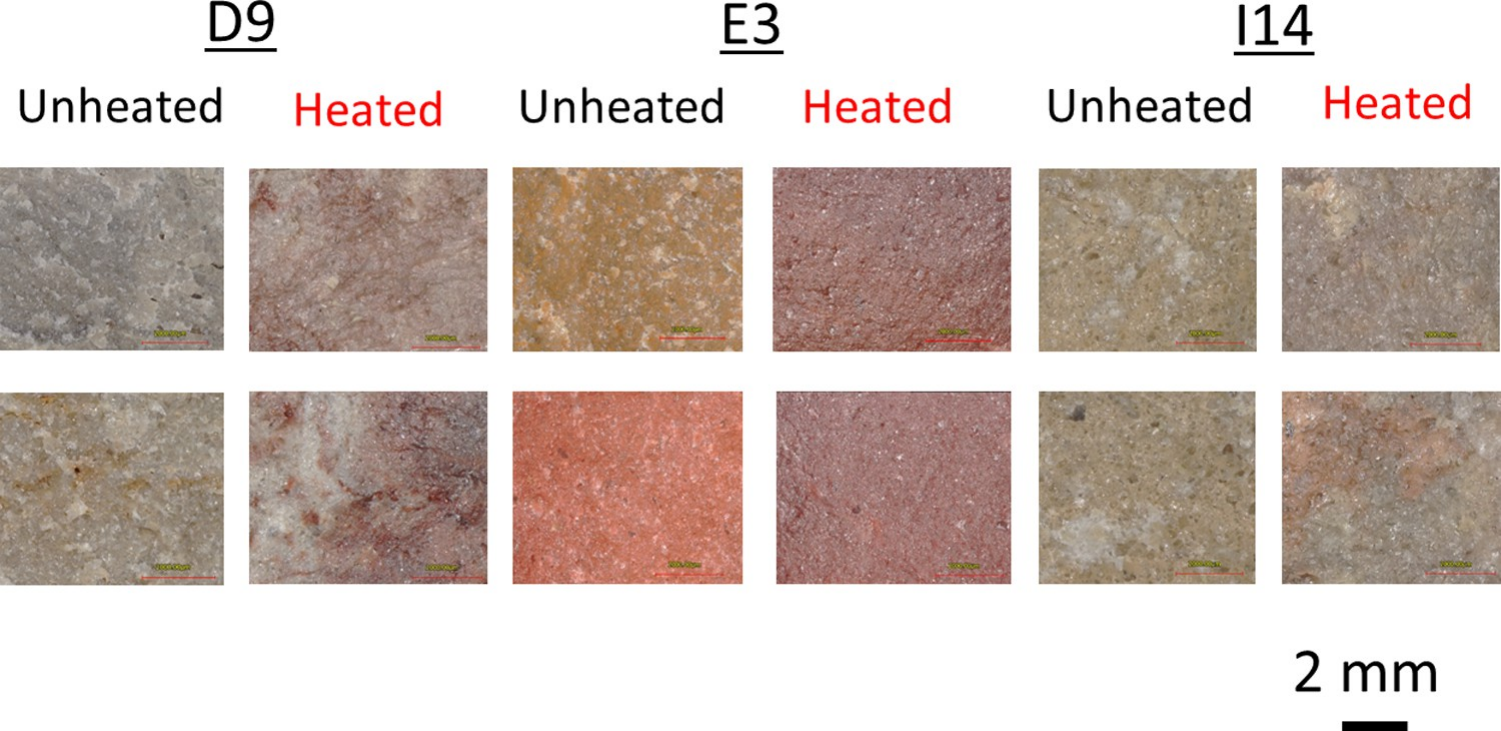
Color and roughness change. Microphotographs of representative silcrete samples from the three silcrete sources used in this study showing variation in color and surface roughness before and after heat treatment.

After heat treatment, all three sources underwent visible changes in roughness and color (Fig 1). In their unheated form, most samples are visibly and tangibly rough, but after heat treatment they become lustrous and smooth. In terms of color, unheated silcrete from source D9 and I14 are mostly light gray and tan whereas E3 is brown to light red. After heat treatment, all sources become noticeably darker and redder.

### Samples and data collection

We randomly selected 49 unheated and 45 heat-treated flakes for analysis from the silcrete knapped by Brown after the heat treatment experiments (Table 1). We used an Ocean Optics (now Ocean Insight) USB 2000 UV-Vis-NIR spectrometer to collect visible light (~380–750 nm) percent reflectance measurements simultaneously with CIE L*a*b* values. These data were collected and processed using the manufacturer’s proprietary software, OceanView. We aimed to record four scans on the dorsal and ventral side (total of 8 scans) of each individual flake. This was done to account for intra-specimen variation in color [20] and replicates the approach used for measuring surface roughness with a 3D microscope [14]. For each specimen, we followed a standard protocol for target selection. The flakes were oriented with the platform down and the flake was divided into four quadrants. The first measurement was taken in the lower left quadrant and the subsequent measurements were taken in each quadrant in a counterclockwise manner (*i*.*e*., lower left, top left, top right, lower right).

**Table 1:** Summary of the samples analyzed in this study and the number of flakes and scans per source.

Source	Block	Treatment	Flakes (n =)	Scans (n =)
D9	D9.1.10A	Unheated	1	8
	D9.1.10B	Heated	5	56
	D9.1.12A	Unheated	5	40
	D9.1.12B	Heated	6	48
	D9.1.12C	Unheated	7	56
	D9.1.12D	Heated	2	24
E3	E3.1.1A	Unheated	6	48
	E3.1.1B	Heated	7	56
	E3.1.5N	Unheated	5	40
	E3.1.5O	Unheated	3	24
	E3.1.5P	Heated	3	24
	E3.1.6A	Heated	5	40
	E3.1.6B	Unheated	5	40
	E3.1.6C	Heated	3	24
I14	I14.2.16A	Unheated	15	120
	I14.2.16B	Heated	16	128
Total	16	-	94	776

Instrument parameters for data collection include the following points. The spectrometer used an Ocean Optics LS-1 series tungsten halogen light source optimized for use in the VIS-Shortwave NIR range (360–2000 nm). The probe was placed in the “diffuse-reflectance” position which forces the light to be emitted at a 45-degree angle to the surface of the stone. In the OceanView software, the reference illuminant used to calculate CIE L*a*b* values was D65, which simulates midday light in Western Europe, because it is the most widely used daylight simulant light source standard for most applications [60]. The probe and probe-holding metal block for the USB 2000 are optimized for use on completely flat specimens; variations in the surface topography of our knapped flakes introduced the possibility of ambient light from the environment interfering with data collection. During each scan, an opaque black plastic party hat was placed over the spectrometer probe and silcrete flake to mitigate interference. We deliberately used a cheap, lightweight, and widely available item for this purpose; an opaque piece of cloth, an overturned bucket, or aluminum foil could all serve the same role. A total of 776 observations were collected on 94 silcrete specimens (flakes). All specimens yielded eight observations each, except for D9.1.10B.6A and D9.1.10B.6B, which have 16 observations each.

We generated summary statistics and conducted predictor (variable) screenings and graphical data exploration using R [61] and JMP Pro 15.0 [62] to better understand variation in color change and to determine which variables were important for discerning unheated and heat-treated silcrete across all three sources. More specifically, we utilized ggplot2 [63], tidyverse [64], and gridExtra [65] packages in R to create our figures. All our raw data, code, and supplementary materials can be found on the OSF repository through this link: https://osf.io/3qkd2/.

## Results

### Colorimetry

Following from our previous work using surface roughness measurements [14], we averaged the colorimetry results for each specimen for our initial data exploration (i.e., the average of all 8 scans for one specimen). This gives each sample one measurement per variable (*i*.*e*., hue-angle, chroma, L*, a*, b*). We did this to make our results easier to compute and visualize, while also attempting to address intra-specimen variation in color which can complicate interpretation and identification of general trends.

**Fig 2:**
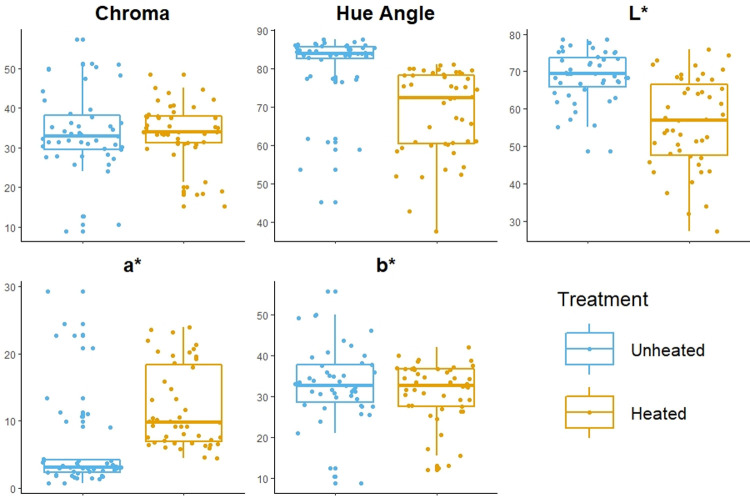
CIE L*a*b* variables across level of treatment. Boxplots comparing the distributions of five colorimetry variables (chroma, hue-angle, L*, a*, b*) between unheated and heat-treated silcrete specimens from all three sources in this study.

The averaged CIE L*a*b* color values, as well as the values for the derived variables chroma and hue-angle, show that some of these variables are more useful than others for identifying heat treatment (Fig 2). The distributions of chroma and b* between level of treatment for all three sources have a considerable amount of overlap and do not differ significantly (chroma: Wilcoxon rank sum test, W = 1026, p = 0.55; b*: Wilcoxon ranked sum test, W = 1201, p = 0.47). However, hue-angle, L*, and a* show promising differences between heated and unheated silcrete. The differences in the distributions of hue-angle between heated and unheated silcrete suggests that unheated silcrete has a yellower color whereas heat-treated silcrete is more orange/red. Further, the overall distributions for L* show that heat-treated silcrete is darker than unheated silcrete. Lastly, the differences in the distributions of a* between unheated and heated silcrete indicates that heat-treated silcrete is more red than unheated silcrete.

**Fig 3:**
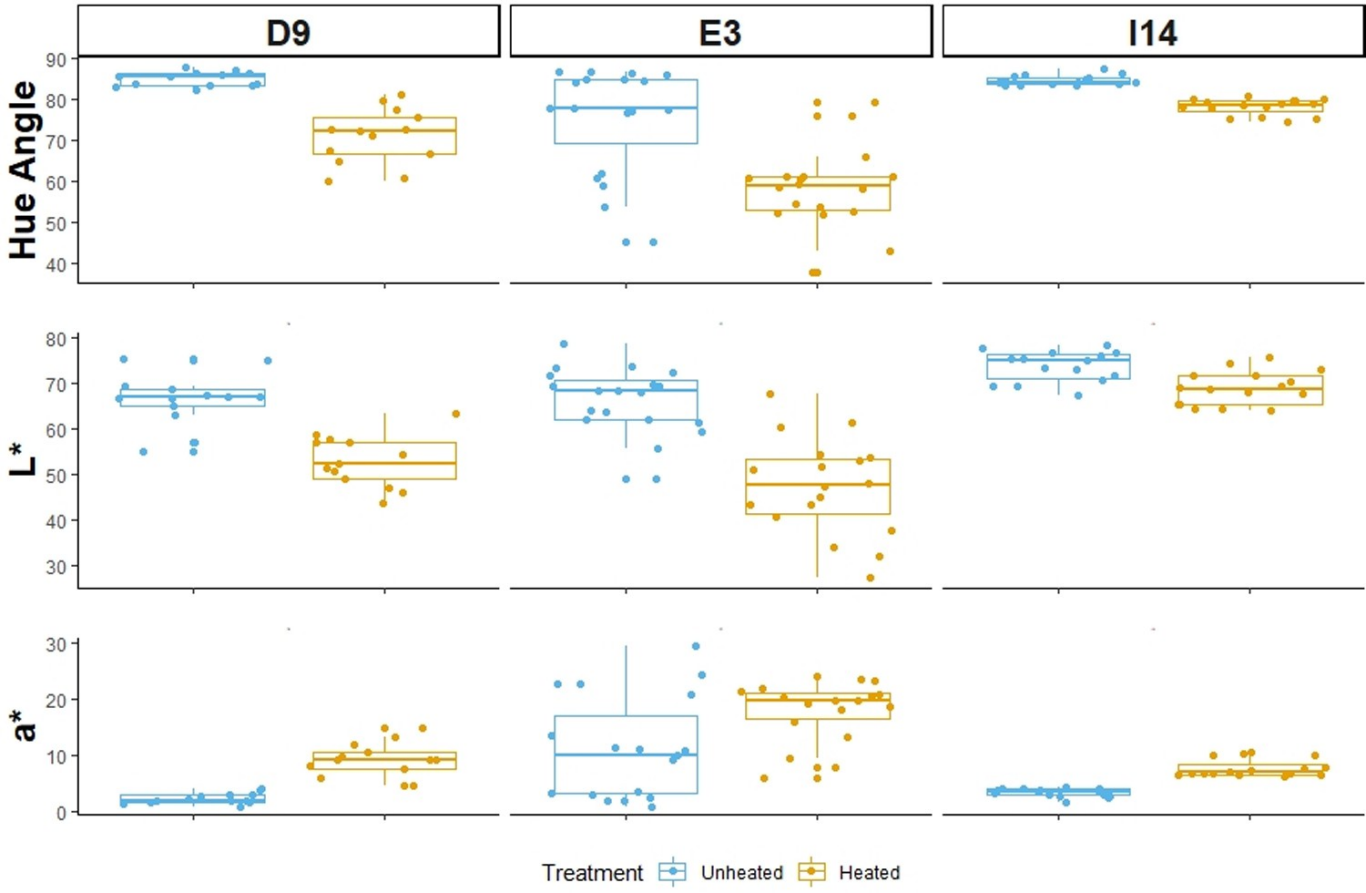
CIE L*a*b* variables across level of treatment by source. Boxplots comparing the distributions of three colorimetry variables (hue-angle, L*, a*) between unheated and heat-treated silcrete specimens across all three sources (D9, E3, I14). Chroma and b* were excluded from this figure because they did not show significant differences between level of treatment.

The patterns exhibited by the distributions of hue-angle, L*, and a* are consistent across all sources (Fig 3). In all three sources (D9-1, E3-1, I14-2), heated silcrete is more red/orange and darker than unheated silcrete (see Table 2 for delta values). However, the range and magnitude of these differences varies between sources in some respects. The range of the distribution of L* for source I14 for both heated and unheated samples is larger than that of E3 and D9. Additionally, the magnitude of difference in lightness between heat-treated and unheated silcrete is smaller for I14. This suggests that source I14 is lighter than both E3 and D9 and that after heat-treatment, silcrete from I14 undergoes less of a change. Further, this pattern is seen in the range of the distribution of a* for source E3 is larger than that of source I14 and D9. This suggests that E3 is generally redder than I14 and D9 and does not change as drastically.

**Table 2:** Colorimetry results by silcrete source and heat treatment state with average CIE L*a*b* and standard deviation from unheated to heated.

Source	Treatment	Av. Chroma (SD)	Av. Hue Angle (SD)	Av. L* (SD)	Av. a* (SD)	Av. b* (SD)
D9	Unheated	25.17 (7.06)	84.97 (2.15)	66.50 (9.52)	2.21 (1.23)	25.06 (7.02)
	Heated	28.51 (8.15)	70.92 (8.65)	52.86 (4.74)	9.06 (4.74)	26.71 (7.80)
	Δ	3.34	-14.05	-13.64	6.85	1.65
E3	Unheated	40.1 (11.80)	75.2 (12.42)	66.33 (9.53)	10.79 (9.04)	37.74 (11.20)
	Heated	35.21 (9.57)	58.14 (9.94)	47.27 (11.75)	17.93 (5.88)	29.82 (9.27)
	Δ	-4.89	-17.06	-19.06	7.14	-7.92
I14	Unheated	34.45 (5.12)	84.64 (2.01)	73.82 (8.52)	3.26 (1.46)	34.28 (5.07)
	Heated	36.56 (5.79)	78.15 (3.32)	69.02 (9.17)	7.62 (2.93)	35.69 (5.45)
	Δ	2.11	-6.49	-4.8	4.36	1.41

**Fig 4:**
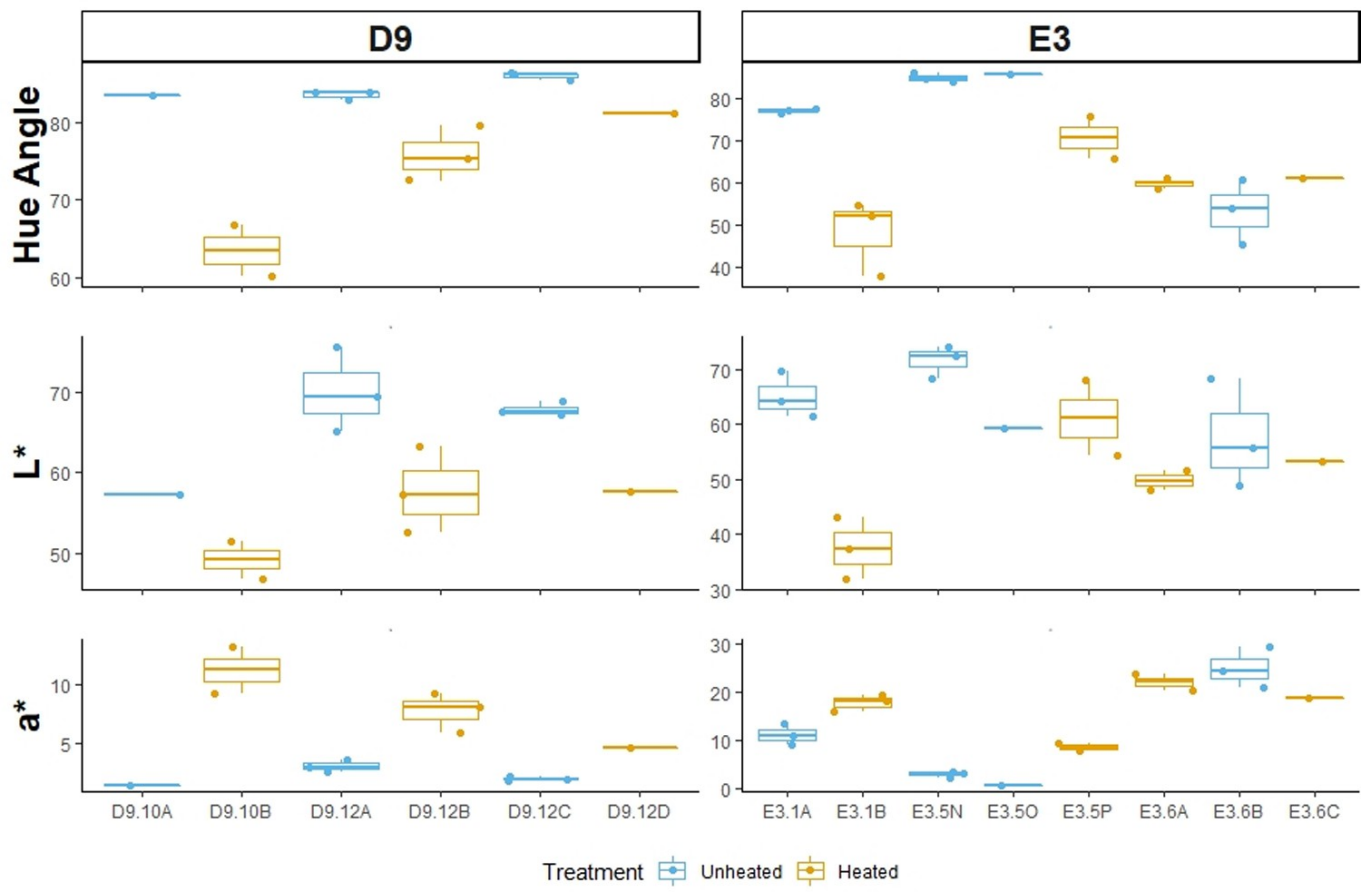
CIE L*a*b* variables across level of treatment by nodule. Boxplots comparing the distributions of three colorimetry variables (hue-angle, L*, a*) between unheated and heat-treated silcrete specimens across sources D9 and E3 and within nodules. Chroma and b* were excluded from this figure because they did not show significant differences between level of treatment. Source I14 was not shown in this figure because it only had one paired nodule and the intra-nodule results can be seen in Fig 3. The naming convention for the nodules seen on the x-axis is Source.Nodule. Thus, D9.10A means that the sample is nodule 10A from source D9.

The patterns in the distributions of hue-angle, L*, and a* observed across sources are similar between nodules of silcrete except for one pairing (Fig 4). We excluded source I14 from this comparison because there was only one paired block from this source so the inter-nodule comparison for this source is the same as the distributions seen in Fig 3. For all paired nodules, except pairing E3.6, heat treatment caused the nodules to become redder and darker. There was little change after heat treatment in E3.6 and these small changes were the opposite pattern seen in the rest of the nodules; the heated nodules became lighter and less red. For pairing E3.5, the results were mixed for L*. One of the unheated nodules showed substantial differences in L* values compared to the heated nodule, but the second unheated nodule was indistinguishable from the heated nodule.

### Visible reflectance spectroscopy data

#### Heated vs unheated spectral differences

**Fig 5:**
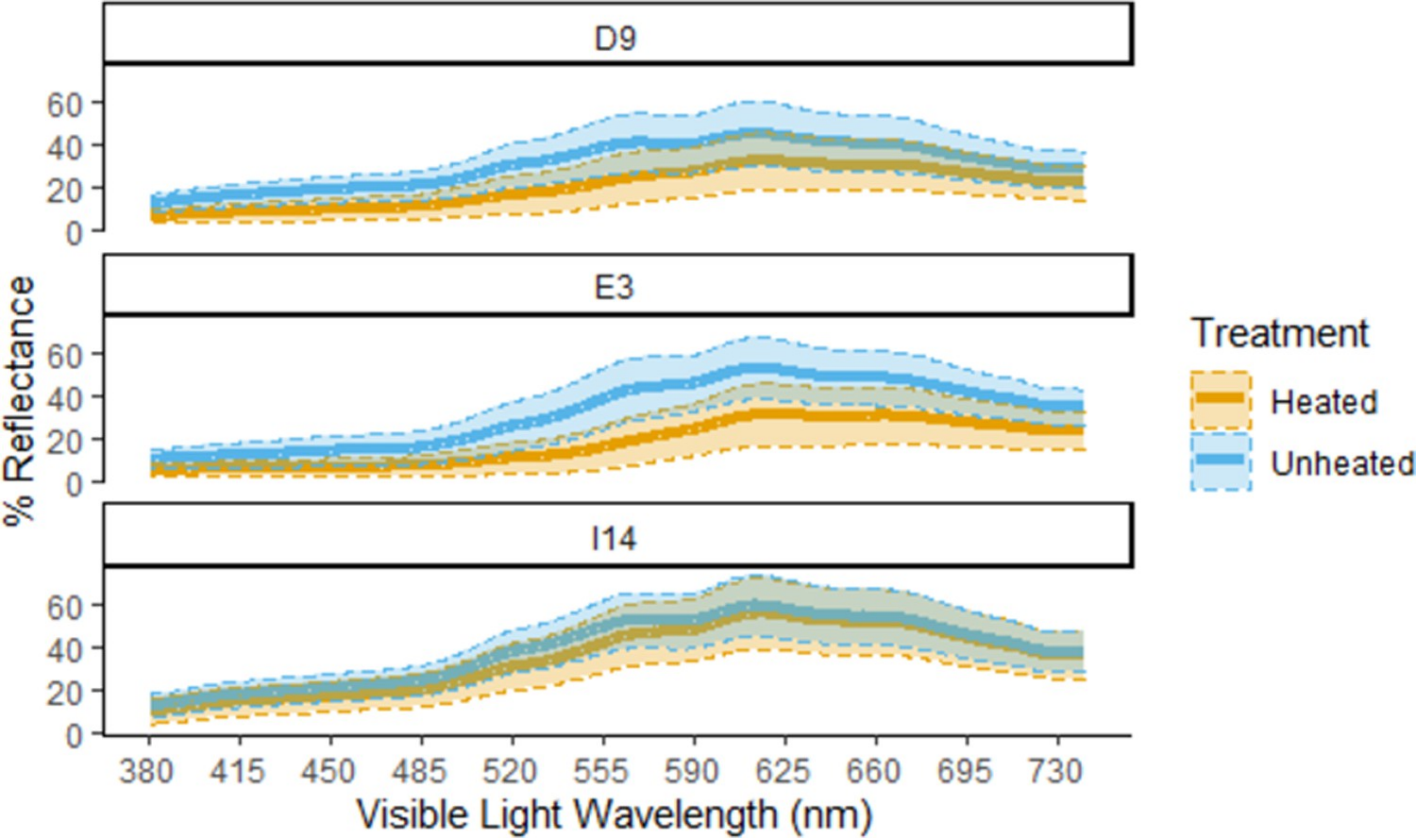
Percent reflectance by level of treatment across sources. Mean percent reflectance (bold lines) results by silcrete source for unheated vs heat-treated silcrete. The shaded blue and yellow areas associated with each line indicate the standard deviation of observed percent reflectance values.

The average spectral reflectance measurements demonstrate that there are differences between unheated and heat-treated silcrete (Fig 5). In all three sources, unheated silcrete has higher percent reflectance values than heat-treated silcrete. Source E3 has the largest difference between unheated and heated measurements while I14 has the smallest difference. The largest differences occur between about 575 and 585 nm which corresponds to the yellow component of the visible light spectrum. In both unheated and heated silcrete, the percent reflectance measurements peak at around 620 nm which is the red region of the visible spectrum, while the lowest values correspond to the blue and purple region of the spectrum.

#### Spectral data processing for statistical analysis

Prior to conducting any statistical interpretation of the spectral reflectance measurements, the data set was subjected to multiple processing steps. Reflectance variables measured spanned ~340 through ~1026 nm, extending beyond the visible spectrum of approximately 380–750 nm. Initial data exploration revealed a substantial number of negative or zero values between ~740 and ~750 nm. Variables below ~380 nm and above ~740 nm were culled from the data set. The resulting data set was composed of 1018 fractional wavelength variables from 380.295 to 740.933 nm, none of which contained zero or negative observation values. The raw data were imported into the chemometrics software package Solo (Eigenvector Research, Inc.) for pre-processing. First, we ran a Savitzky-Golay, or SavGol, filter algorithm [66] to generate a derivative from the raw data. The SavGol algorithm is a low-pass filter that fits a low-degree polynomial to the raw data using the linear least squares method to attenuate high-frequency noise through data smoothing; it has been widely used in instrumental analytical chemistry and other fields for decades [67]. Filter parameters were as follows: Filter Width (*w*) = 9, Polynomial Order (*o*) = 2, and Derivative Order (*d*) = 1. Next, we ran the autoscale function on the first derivative data generated by the SavGol algorithm. The Solo autoscale function uses mean-centering followed by dividing each column (variable) by the standard deviation of the column. The offset parameter was set to the default of 0. This yielded the processed data set on which all subsequent analyses were conducted.

The processed data set was then imported to JMP Pro 15. We ran the Multivariate *k-*Nearest Neighbor (*k-*NN) Outliers module using the default setting of *k* = 8, which generates Euclidean distance-to-neighbor plots for *k* of 1 through 8. *k-*NN outlier detection uses the simple premise that observations distant from a specified number of nearest other observations are likely to be outliers. We elected to base our identification of multivariate outliers on the results from *k = 3* (*i*.*e*., the three nearest neighbors to each point). Ultimately, 30 outlier observations were identified for exclusion from the data set (~3.9% of observations), leaving 746 observations remaining for statistical analysis. The data sets resulting from each stage of processing described above are provided as separate tabs in S1 Table.

### Model results

#### Classification of heat treatment state using reflectance spectroscopy data

*k-*Nearest Neighbors classification is a supervised non-parametric machine learning algorithm first proposed by Fix and Hodges [68] that makes categorical predictions based on proximity in multidimensional feature space. While distance to nearest neighbors was used to identify outliers in the previous section, here a prediction about group membership was made for an observation based on the majority group identity of a specified number of nearest neighbor observations using Euclidean distance. For example, if *k* = 1, the group identity of the nearest point to an observation is used to predict the group classification of that observation. Even numbers of *k* should be avoided because they permit ties in two-group classification problems. For *k* = 2, if the two closest neighbors to an observation belong to different groups, then the model will randomly select the predicted group. Larger odd values of *k* permit majority voting and result in a model that will generalize better to novel data. In order to select the optimal value of *k*, the training data set is used to make predictions for the validation set at multiples values of *k*. Despite the use of a training data set, in contrast to a method like linear discriminant analysis *k-*NN does not actually have a training period during which a discriminative function is calculated. Instead, *k-*NN references the training data to make real-time predictions for each observation, hence the term “lazy learning” to describe this and related methods [69]. Misclassification rates for the validation set are calculated and the analyst selects a value of *k* which minimizes the misclassification rate and uses that model to make predictions for the test set. The test set is used to assess how the model performs on novel input data after optimization with the validation set. Here, we applied *k-*NN to the processed data described in the previous section to solve a binary classification problem: heat-treated or unheated silcrete. Both *k-*NN models that follow used all 1018 first derivative percent reflectance variables, without any dimension reduction, as predictors for the categorical response of heat treatment state.

In JMP Pro 15, we sub-divided our processed spectral reflectance data into training, validation, and test subsets using a nominal 70%/15%/15% split. Observation role was assigned in a random, stratified, grouped manner. Observations were grouped by specimen ID number in order to ensure that all measurements of a given silcrete flake were associated with one of the subsets. This was important because in a functional application with archaeological artifacts, all measurements of a single flake would be assigned to the unknown category (comparable to the test set here). The observations were also stratified by heat treatment state. Thus, an approximately equal number of observations in each of the three sets came from the heated and unheated categories, and all observations associated with a given flake were placed in the same data set (training, validation, or test). Observation counts for *k-*NN model A are shown below in Table 3.

**Table 3:** k-NN model A observation roles for training, validation, and testing.

	Treatment State		Silcrete Source		
Row Role	Heated	Unheated	D9	E3	I14
**Training**	307	277	186	203	195
**Validation**	39	39	16	32	30
**Test**	40	44	22	54	8

The second classification analysis also used a *k-*NN approach (model B). However, in this case the training, validation, and test set was stratified by silcrete source and not by treatment state, while still grouped by specimen ID as before. JMP does not permit stratification by more than one category at a time if a grouping category is also being used. Comparing model A to a model in which the sets were stratified by source was necessary to investigate the sensitivity of this approach to information available to and decisions made by the analyst during model construction. The processed derivative observations were again nominally divided into 70% training, 15% validation, and 15% test subsets. Observation counts for *k-*NN model B are shown in Table 4 below.

**Table 4:** k-NN model B observation roles for training, validation, and testing.

	Treatment State		Silcrete Source		
Row role	Heated	Unheated	D9	E3	I14
**Training**	309	277	178	220	188
**Validation**	30	44	22	30	22
**Test**	47	39	24	39	23

For *k-*NN model A, we elected to use a hyper-local model with *k* = 1 (S2 Table). Although *k* = 2 actually yielded a one fewer misclassified observation in the test set, we did not give this any credence because an even value of *k* introduces an element of randomness into predictions. The observation-level misclassification results for model A are provided in Table 5 below. We do not include training set misclassification rates here because they are irrelevant to interpreting model effectiveness. These results and their associated low misclassification rates are indicative of the predictive power of the *k-*NN approach to heat treatment detection, but they are not strictly relevant to a real-world application.

**Table 5:** k-NN model A observation-level prediction results.

Actual Treatment	Predicted Treatment		% Misclassified
**Validation Observations**			
	Heated	Unheated	
Heated	39	0	0.0%
Unheated	1	38	2.6%
**Test Observations**			
	Heated	Unheated	
Heated	39	1	2.6%
Unheated	3	41	7.3%

In a study of archaeological silcrete, the objective must be to classify each artifact as heat-treated or unheated; the prediction for each replicate measurement of an artifact is not anthropologically relevant. For that reason, we reinterpreted our results at the specimen-level by using the majority prediction for each specimen to assign heat treatment state (*e*.*g*., 5/8 observations for one flake classified as heated predicts heated for the flake; a 4/4 tie yields an unassignable specimen). As illustrated by Table 6 below, extending the principle of majority voting from *k-*NN classification of observations to the specimen level is highly effective and representative of how this approach would be applied to an actual archaeological assemblage. Model A yielded zero misclassified specimens in the validation and tests sets, and only a single unassignable specimen out of 11 flakes in the test set. In retrospect, it may seem an obvious error to have not collected an odd number of observations for each specimen to prevent ties; the majority voting approach to artifact classification was not foreseen when data collection began.

**Table 6:** k-NN model A specimen-level prediction results.

Actual Treatment	Predicted Count			% Misclassified	% Not assignable
**Validation Specimens**					
	Heated	Unheated	Not Assigned		
Heated	5	0	0	0.0%	0.0%
Unheated	0	5	0	0.0%	0.0%
**Test Specimens**					
	Heated	Unheated	Not Assigned		
Heated	5	0	0	0.0%	0.0%
Unheated	0	5	1	0.0%	16.7%

For model B, in which the sets were stratified by geological sources, we obtained largely comparable results to model A. In this case, we chose a *k-*NN model that was far less local than model A, using *k* = 7 (Table 7). The validation set first reached its minimum number of observation misclassifications (11/74) at *k* = 4; however, the test set achieved minimum misclassification at *k* = 7 (5/86). Using observation predictions from the *k* = 7 model (S2 Table) to assign specimen-level classification, again based on the majority prediction for each flake, no specimens were unassignable and only a single validation set specimen was misclassified (false positive; unheated predicted as heated). The results from these two models indicate that the -*k-*NN classification approach used with visible reflectance spectroscopy data is a viable method of investigating the heat treatment of silcrete and does not require *a priori* knowledge of geological source. This last point is critical to reducing the costs and invasiveness of such a study by removing the need for geochemical characterization of artifacts and source samples for provenience analysis prior to undertaking a heat treatment investigation.

**Table 7:** k-NN model B specimen-level prediction results.

Actual Treatment	Predicted Count			% Misclassified	% Not assignable
**Validation Specimens**					
	Heated	Unheated	Not Assigned		
Heated	4	0	0	0.0%	0.0%
Unheated	1	5	0	16.7%	0.0%
**Test Specimens**					
	Heated	Unheated	Not Assigned		
Heated	6	0	0	0.0%	0.0%
Unheated	0	5	0	0.0%	0.0%

The low misclassification rates at the observation and specimen levels for both models A and B are striking considering that the underlying variables were not optimized for their effectiveness in predicting heat treatment state; we simply used all 1018 SavGol filtered percent reflectance variables. We adopted a naïve approach to variable selection to investigate how user-friendly *k-*NN is for this application and to learn how much improvement in misclassification rate is possible through predictor screening and/or dimension reduction. *k-*NN models A and B also seem to have escaped the “curse of dimensionality” [70, 71], in which the distance between nearest neighbor and farthest neighbor converges in some high-dimensionality datasets, rendering the *k-*NN query unstable. In complementary attempts at applying *k-*NN to our data, we explored running the classification algorithm on principal components derived from the SavGol smoothed reflectance variables. For example, using the same division of training, validation, and test sets as in model A, *k-*NN was run using just three principal components which accounted for 82.7% variance in the data set. The model optimized at *k* = 5, yielding a validation set overall misclassification rate of 11.5% and test set misclassification rate of 5.9% for observation-level predictions, substantially worse than model A. This was the best outcome from applying *k-*NN to dimension reduced data and we subsequently abandoned the use of principal components.

The full data set of 746 observations for 1018 reflectance variables was next subjected to predictor screening (*i*.*e*., feature selection) in JMP Pro 15 to identify a subset of variables likely to be particularly informative for categorical classification of heat treatment state. Predictor screening relies on bootstrap forest partitioning (called decision trees in JMP) to identify variables that may have a significant effect on predicting a response on their own or in combination. Using the output of predictor screening with 1000 decision trees we selected the top ~5% (50) of reflectance variables. We then ran a new *k-*NN analysis with just those predictors, using the same training, validation, and test subsets as model A (stratified by heat treatment state, observations grouped by specimen). We repeated this procedure using the top 40, 30, 20, and 10 predictors. None of the five predictor-screened variants of *k-*NN model A outperformed the original 1018 reflectance variable model on the basis of test set misclassification rate. This indicates that, at least for the three South African sources considered here, rigorous predictor screening is not an essential step to classification of heat treatment state in silcrete when using visible reflectance spectroscopy. The five predictor screening analyses did yield one interesting result: 15 strongly correlated variables between 580.884 nm and 585.825 nm consistently appeared among the highest ranked predictors. This narrow range corresponds to yellow light (~560–590 nm) and we explore the role of CIE L*a*b* color variables for predicting heat treatment in the next section.

In a final attempt to assess whether predictor screening can lead to lower misclassification rates, we used another 1000 decision tree round of screening to select the top ~25% (254) of variables and ran a nominal logistic regression for treatment state. The 746-observation data set was randomly divided into training and validation subsets using a 75%/25% split, with observation role stratified by heat treatment state and grouped by specimen. A test set was not used because the goal was to assess the predictive importance of variables and not to build an optimized discriminant model that can generalize well to novel data. The 254 selected variables were loaded to a stepwise nominal regression module in JMP. The module automatically added variables (forward stepwise) until the optimal model was identified based on the parameter Max Validation RSquare, defined as

$$RSquare\;Validation=1-\frac{{SSE}_{Validation}}{{SST}_{Validation}}$$

where *SSE*_*Validation*_ is the squared, summed prediction error for the validation subset and *SST*_*Validation*_ is the squared and summed differences between the actual responses in the validation set and their mean.

A model using just nine variables was identified as optimal: 418.419 nm, 435.478 nm, 437.697 nm, 453.19 nm, 459.073 nm, 460.91 nm, 487.606 nm, 518.775 nm, 605.852 nm. Notably, these variables exclude all of the 15 variables in the ~580–585 nm range mentioned above. The nine identified variables were further pared down manually by eliminating variables in ascending order by LogWorth (-log10(p-value)), a measure of each variable’s significance to the model, and observing the effect on validation set misclassification rate at each step. Ultimately, the variables 435.478 nm, 437.697 nm, 459.073 nm, 460.91 nm, 487.606 nm, and 518.775 nm yielded the lowest overall validation set misclassification rate for a nominal logistic regression model: 13.9%. As illustrated by the observation-level misclassification rates and the specimen-level misclassification rates in the two tables below (Tables 8 and 9), this model has a greater false positive issue than either of the *k-*NN models.

**Table 8:** Nominal logistic regression model observation-level prediction results.

Actual Treatment	Predicted Treatment		% Misclassified
**Validation Observations**			
	Heated	Unheated	
Heated	88	6	6.4%
Unheated	18	61	22.8%

**Table 9:** Nominal logistic regression model specimen-level prediction results.

Actual Treatment	Predicted Count			% Misclassified	% Not assignable
**Validation Specimens**					
	Heated	Unheated	Not Assigned		
Heated	12	0	0	0.0%	0.0%
Unheated	3	8	0	27.3%	0.0%

Examination of the specimen-level validation set found that the three misclassified flakes (unheated predicted to be heated) all derive from the same silcrete nodule (6B) collected from source E3. Furthermore, the two misclassified specimens in the training set also derive from nodule 6B. This suggests that a single piece of silcrete with anomalous properties relative to other nodules from that source underlies all of the false positive prediction error in this model. The atypical response of E3.6B to heating was noted earlier. Interestingly, in model A, which like the nominal logistic regression used a validation set stratified by treatment state and not source, four of the five specimens from nodule 6B were assigned to the training set while the fifth was assigned to the test set where its observation predictions tied 3/3 leaving the specimen unassignable. Of the 30 observations identified as multivariate outliers during data processing, six are associated with three different flakes from nodule 6B.

#### Classification of heat treatment state using colorimetry data

After completing statistical interpretation of the reflectance spectroscopy results, we attempt to reproduce the success of the predictive *k-*NN models A and B using colorimetry data. In contrast to the reflectance spectroscopy data set, the colorimetry data set is low dimensionality and consists of just the L*, a*, and b* variables. With regard to instrumentation, reflectance spectrometers are capable of collecting both spectroscopy and colorimetry data (CIE L*a*b* variables are derived from spectroscopy measurements), while dedicated colorimeters generally do not permit collection of wavelength-scale reflectance data. The objective of attempting to achieve discrimination of heated and unheated silcrete using colorimetry data collected during this study was to investigate if silcrete heat treatment classification using less expensive and more robust field-portable colorimeters is a viable path for future method development.

Analysis of the colorimetry data proceeded in largely the same manner as the reflectance spectroscopy data, but without the initial data processing steps (SavGol filtering, centering, and scaling). The colorimetry observations and the prediction results from the associated *k-*NN models may be found in S3 Table. In order to be comparable to the spectroscopy data sets, 30 outlier observations were identified and excluded from the colorimetry data set using Multivariate *k-*NN Outlier analysis with *k* = 3, leaving 746 observations for analysis. *k-*NN model C was constructed as the colorimetry complement to model A. The model C observations had their validation role randomly assigned, stratified by heat treatment state, and grouped by specimen ID with a training, validation, and test subset nominal split of 70%/15%/15%. Model C observation-level misclassification rates optimized at *k* = 7 for the validation set and *k* = 5 for the test set. Table 10 below shows the observation-level predictions and misclassification rates for model C at *k* = 5.

**Table 10:** k-NN model C observation-level prediction results.

Actual Treatment	Predicted Treatment		% Misclassified
**Validation Observations**			
	Heated	Unheated	
Heated	32	8	25.0%
Unheated	3	37	8.1%
**Test Observations**			
	Heated	Unheated	
Heated	33	4	12.1%
Unheated	5	40	12.5%

Using the same majority voting approach as for the spectroscopy models, at the specimen-level *k-*NN model C (Table 11) performed slightly worse than its spectroscopy twin model A. Model C yielded one misclassified validation set specimen (relative to zero misclassified in model A) and a single unassignable test set specimen (comparable to model A). The single misclassified specimen was a false negative error; a heated flake was predicted as unheated.

**Table 11:** k-NN model C specimen-level prediction results.

Actual Treatment	Predicted Count			% Misclassified	% Not assignable
**Validation Specimens**					
	Heated	Unheated	Not Assigned		
Heated	4	1	0	20.0%	0.0%
Unheated	0	5	0	0.0%	0.0%
**Test Specimens**					
	Heated	Unheated	Not Assigned		
Heated	5	0	0	0.0%	0.0%
Unheated	0	5	1	0.0%	16.7%

As the analog to spectroscopy model B, we lastly constructed a *k-*NN model with colorimetry observations assigned to training, validation, and test sets (70%/15%/15%) randomly, stratified by silcrete source and grouped by specimen ID. The same 30 outliers were removed as in model C prior to observation role assignment. For model D, the validation set misclassification rate optimized at *k* = 2 (9 misclassified observations), which yielded a test set misclassification count of 11 observations. In light of the previously noted random aspect of using even values of *k* in a two-class model, we selected *k* = 3 which produced the results in the Table 12 below.

**Table 12:** k-NN model D observation-level prediction results.

Actual Treatment	Predicted Treatment		% Misclassified
**Validation Observations**			
	Heated	Unheated	
Heated	43	10	23.3%
Unheated	2	20	10.0%
**Test Observations**			
	Heated	Unheated	
Heated	28	3	10.7%
Unheated	3	50	6.0%

At the specimen level using majority prediction for classification, model D performed similarly to and arguably better than spectroscopy model B (Table 13). Both models yielded zero misclassified or unassignable specimens in their respective test sets. Model B had a single misclassified validation set specimen, whereas model D had single unassignable validation set specimen.

**Table 13:** k-NN model D specimen-level prediction results.

Actual Treatment	Predicted Count			% Misclassified	% Not assignable
**Validation Specimens**					
	Heated	Unheated	Not Assigned		
Heated	6	0	1	0.0%	14.3%
Unheated	0	3	0	0.0%	0.0%
**Test Specimens**					
	Heated	Unheated	Not Assigned		
Heated	4	0	0	0.0%	0.0%
Unheated	0	7	0	0.0%	0.0%

## Discussion

Recent work has focused on improving the ability of archaeologists to detect heat-treated artifacts using quantitative methods in order to improve reproducibility of results across researchers [14–16, 28, 30]. Our study builds on these approaches by exploiting an observable phenomenon in heat-treated stone: color change. We utilize colorimetry and reflectance spectroscopy in combination with machine learning to classify heat-treated and unheated silcrete. Our results, while preliminary and based on a limited number of silcrete sources, suggest that quantified measures of color can be reliable predictors of heat treatment when incorporated into *k*-NN machine learning models.

Our *k-*NN models are comparable in accuracy to the surface roughness method [14] and the visual identification of heated silcrete by expert analysts [13]. However, if we utilize our method with a majority-voting approach to specimen-level classifications, we can improve prediction accuracy significantly (Table 14). In some cases, ties in the observation-level predictions prevented us from assigning a level of treatment to a specimen. Unassignable flakes occurred more often with unheated silcrete than heat-treated silcrete. For the future, a potential simple solution to this issue is collecting an odd number of observations per flake (*i*.*e*., seven or nine scans per flake instead of eight). Some flakes from one nodule in particular, E3.6B, were unassignable in both Model A (S2 Table: Model A predictions by specimen–test set) and Model C (S3 Table: Model C predictions by specimen–training set). One possible cause of this issue is that silcrete from source E3 in its natural unheated state is redder than other silcretes sampled in this study so the change in redness after heating is less pronounced which may be leading to some observations being misclassified. In future work, the combination of surface roughness measures and colorimetry or reflectance spectroscopy data may be able to resolve the classification of such materials.

**Table 14:** For specimen-level predictions, the test set of all four k-NN models yielded no misclassified flakes. This suggests that our visible spectrum approach to heat treatment detection is robust and future work to develop a CIE L*a*b*-only method using less expensive and more rugged dedicated colorimeters holds promise.

			Actual Treatment	
		Test Set Specimen-level Predictions	Heated	Unheated
Reflectance Spectroscopy *k-*NN models with 1018 variables	Model A: *k* = 1	% Misclassified	0.0%	0.0%
		% Unassignable	0.0%	16.7%
	Model B: *k* = 7	% Misclassified	0.0%	0.0%
		% Unassignable	0.0%	0.0%
Colorimetry *k-*NN models using L*, a*, and b*	Model C: *k* = 5	% Misclassified	0.0%	0.0%
		% Unassignable	0.0%	16.7%
	Model D: *k* = 3	% Misclassified	0.0%	0.0%
		% Unassignable	0.0%	0.0%

Our colorimetry results support previous studies which show that heat treatment causes an increase in redness of raw materials [1, 17, 18, 20, 72]. However, the more nuanced percent reflectance data suggest that much of our ability to discern heat-treated and unheated silcrete is due to differences in the yellow component of the visible light spectrum. Further, our results show that silcrete from these three sources become darker after heat treatment. At first glance, this seems to contrast with post-depositional studies of heated chert that show an increase in lightness as chert is heated at higher temperatures [22], which suggests that patterns of color change may not be generalizable across raw material types. However, Schmidt and colleagues [20] have shown that around 200° C silcrete begins to become redder in two sources from Botswana and one source from South Africa. At around 300° C these samples reach maximum redness and overall redness starts to decline after 450°C. Overall, the four sources they analyzed increased in “redness” by around 7–9%. This suggests that silcrete heated to above 350°C may become lighter in color after the initial reddening and darkening, which is similar to the results found by Fiers and colleagues [22] with chert. One possible path to addressing temperature dependent variation in color properties through future experimental work is to apply *k*-NN regression instead of *k*-NN classification in order to predict maximum heating temperature rather than a binary class label.

It is important to note that since Fiers and colleagues [22] primary goal was not to understand changes occurring during intentional heat treatment of raw materials, our results may not be directly comparable. Additionally, our study used silcrete that was only heated to one temperature (350° C) in order to limit the range parameters under consideration in this proof-of-concept study. Further, our samples exhibit no crazing, blackening, or other thermally induced features. If silcrete was heat-treated in an open-fire or using the ember method, it is highly likely that our samples would have significantly more variation in color change due to uneven heating across samples which may impact the colorimetry results.

The variation seen in redness and lightness in heated raw materials at different temperatures suggests that an approach based on color, like the one described here, may not only be an indicator of heat treatment but may also be able to discern the process by which it was heated. We intend to address this issue through actualistic heat treatment experiments in a future study as a prerequisite to applying our classification system to an archaeological assemblage. Additionally, this will allow us to better understand how adhering substrate and organic matter from the heat treatment process may influence the colorimetry measurements. Ultimately, our goals are to use this approach to better understand early evidence of heat treatment within the Pinnacle Point site complex on the south coast and to demonstrate a method that may be adapted for use by other researchers.

Another important result of this study is that our approach is generalizable across all three silcrete sources (D9, I14, E3). Models B and D, which stratified the data subsets by source, did not yield superior specimen-level misclassification rates to models A and C, which stratified by heat treatment state. Despite this encouraging result, we want to stress that these models would not be applied to archaeological material unless the applicable geological source was known and included in the experimental data used for model construction. The models presented in this study were created for investigative purposes to better understand how knowledge of source may affect classification accuracy. These results suggest that in the future, such a model has the potential to be applied without knowing the geological source of the raw material, but more studies of regional and global silcrete variation are needed to confirm this.

In addition to our approach’s high accuracy in identifying heat-treated silcrete, we think that there are other benefits that make it useful, particularly the colorimetry aspect of our work. There is a wide range of colorimeters that can be used to collect the data required to apply this method; some of these devices are fairly inexpensive (~$350 USD). Further, this technique is highly portable and non-destructive which means that it can easily be applied in a field research environment by non-experts. It may ultimately be possible to utilize our approach on-site at excavations and even during pedestrian surveys, making documenting heat treatment in both academic and cultural resource management (CRM) projects more feasible.

Conservatively, we think that our new approach can be used as a stand-alone method to identify heat-treated silcrete on the south coast of South Africa. Our data collection and predictive classification technique can be expanded to other regions, and likely to other raw materials, creating multiple avenues for future research. Alternatively, this method could be used in conjunction with other non-destructive methods such as the 3D surface roughness method which uses silicon peels of the surface of artifacts to mitigate the need to export artifacts for destructive analyses [14]. We recommend combining the 3D surface roughness method with the approach described here because it provides the analyst with two independent proxies for heat treatment–similar to the approach taken by Bachellerie and Schmidt [16] in which they compare surface roughness and near-infrared spectroscopy. Utilizing two approaches provides the ability to compare their results and potentially to combine both types of observations into a single predictive statistical model.

## Conclusion

Accurately identifying heat-treated silcrete in the archaeological record is an important endeavor due to the claims made about its relationship to complex cognition [12, 25, 26]. This research tested a new non-destructive, quantitative, replicable, and portable instrumentation approach using colorimetry and visible reflectance spectroscopy to identify heat treatment in three geological sources from South Africa. Our study quantifies color changes in silcrete due to intentional heat treatment and operationalizes those data to predict heat treatment condition. Notably, *k-*NN classification models seem to be sparsely used in archaeometry but yielded considerable success here, with minimal data exploration and transformation required to optimize prediction outcomes. Although this method development study relied on a limited sample of silcrete that does not reflect the considerable diversity of this material worldwide, or even regionally, our findings suggest that reflectance spectroscopy and colorimetry approaches are worth pursuing in South Africa and beyond for archaeological application. The production of study area-specific training data sets for model construction will prove key to the viability of these methods.

## Supporting information

S1 Table Spectral data processing. (XLSX)

S2 Table k-NN Vis spectroscopy. (XLSX)

S3 Table k-NN CIELab. (XLSX)
